# Modeling the Evolution of Riparian Woodlands Facing Climate Change in Three European Rivers with Contrasting Flow Regimes

**DOI:** 10.1371/journal.pone.0110200

**Published:** 2014-10-16

**Authors:** Rui P. Rivaes, Patricia M. Rodríguez-González, Maria Teresa Ferreira, António N. Pinheiro, Emilio Politti, Gregory Egger, Alicia García-Arias, Felix Francés

**Affiliations:** 1 Forest Research Center, Instituto Superior de Agronomia, Universidade de Lisboa, Lisbon, Portugal; 2 CEHIDRO, Instituto Superior Técnico, Universidade de Lisboa, Lisbon, Portugal; 3 Environmental Consulting Klagenfurt, Klagenfurt, Austria; 4 Research Institute of Water and Environmental Engineering, Universitat Politècnica de València, Valencia, Spain; University of Colorado, United States of America

## Abstract

Global circulation models forecasts indicate a future temperature and rainfall pattern modification worldwide. Such phenomena will become particularly evident in Europe where climate modifications could be more severe than the average change at the global level. As such, river flow regimes are expected to change, with resultant impacts on aquatic and riparian ecosystems. Riparian woodlands are among the most endangered ecosystems on earth and provide vital services to interconnected ecosystems and human societies. However, they have not been the object of many studies designed to spatially and temporally quantify how these ecosystems will react to climate change-induced flow regimes. Our goal was to assess the effects of climate-changed flow regimes on the existing riparian vegetation of three different European flow regimes. Cases studies were selected in the light of the most common watershed alimentation modes occurring across European regions, with the objective of appraising expected alterations in the riparian elements of fluvial systems due to climate change. Riparian vegetation modeling was performed using the *CASiMiR-vegetation* model, which bases its computation on the fluvial disturbance of the riparian patch mosaic. Modeling results show that riparian woodlands may undergo not only at least moderate changes for all flow regimes, but also some dramatic adjustments in specific areas of particular vegetation development stages. There are circumstances in which complete annihilation is feasible. Pluvial flow regimes, like the ones in southern European rivers, are those likely to experience more pronounced changes. Furthermore, regardless of the flow regime, younger and more water-dependent individuals are expected to be the most affected by climate change.

## Introduction

For decades scientists have been raising awareness about ongoing global climate change brought about by anthropic greenhouse gas (GHG) emissions into the atmosphere (e.g. [1], [2], [3], [4]). While at first it was possible to raise doubts in relation to the alleged global climate change process, the development and continued improvement of global circulation models (GCM) has allowed the scientific community to project with a high level of confidence that global mean surface temperature will increase over the course of the 21^st^ century [5]. What is more, this trend will be followed by an increase in global averaged mean water vapor, evaporation and precipitation [6]. In Europe, regional circulation models (RCM) forecast climate warming above the projected global mean temperature rise, with precipitation pursuing contrasting tendencies according to region and season [7]. In Northern Europe, annual rainfall is expected to increase, while the opposite trend is expected for southern Mediterranean areas [8]. Nevertheless, seasonal precipitation estimates in these regions are not straightforward. If winter precipitation in northern and central Europe is very likely to rise, in southern Europe there are some uncertainties, with different rainfall projections depending on the emissions scenario. On the other hand, it is consensual that summer rainfall will decrease all over Europe, and the same is true for snow, which is predicted to decrease throughout this continent [9].

Such meteorological changes will significantly affect European river flow regimes, essentially through more pronounced low flow magnitudes in the Mediterranean climate zone and major modifications in high flow magnitudes in snow climates [10]. In summer, higher temperatures and evaporation rates, combined in a number of cases with less precipitation, will reduce runoff in many European regions [11], [12], [13]. Even in nival or glacier-affected basins, runoff is expected to decrease due to a decline in melt water [14], leading to important reductions in floodplain inundations in the summer season. In contrast, higher runoff values in the wet season can enhance the risk of flooding caused by increased heavy rain events in a Mediterranean climate, or sleet (commonly known as “rain on snow events”) in snow ones [5]. This will be further aggravated by the likelihood that modifications in river flow regimes and their associated ecosystems will be amplified by future climate change interactions with anthropogenic pressures, such as increased water withdrawals to satisfy human needs [15], [16].

Rivers have a natural flow regime, on the basis of which aquatic and riparian communities have evolved in reliance on the ecological integrity of their ecosystems [17]. Flow regime alterations can thus have numerous impacts – geomorphological [18], ecological [19] and biological [20] – on those communities. Depending on the severity of changes, it may be that thresholds will eventually be crossed with unforeseeable consequences for mankind [21], given that ecosystems provide ecological services that are critical to the functioning of Earth's life-support system and give a very important contribution to human welfare [22].

Riparian ecosystems are particularly vulnerable to flow regime changes [23], since they are governed mostly by that regime and its stream flow components [24], [25], [26]. Riparia forms a transitional boundary that connects aquatic and terrestrial communities [27], [28], [29], consequently presenting high biodiversity and production [28], [30] while simultaneously harboring the most endangered ecosystems on earth [31], [32]. Additionally, riparian areas perform important hydrologic, geomorphic and biological functions to a greater degree than upland areas, considering the proportional area they cover within a watershed [29]. Indeed, researchers have documented several benefits to freshwater environment occasioned by the presence of riparian vegetation (e.g. [33], [34]), as well as evidence of the effects of its deterioration on instream species [35]. Riparian ecosystems also provide goods and services that are directly valued by human societies, such as reductions in damage from floodwaters [36], [37], supplying suitable areas for bird watching, wildlife enjoyment and game hunting [38], [39], [40], or providing fish for food and recreation [41], [42]. Thus, if decision-makers want to ensure that river restoration and administration produce successful results, they must consider riparian management to be an emerging environmental issue that plays an essential role in water and landscape planning.

Given that flood cycles are paramount in influencing riparian forest patterns [43], new tools are urgently needed to provide a long-term quantification of the predictable effects of stream hydrological re-setting on riparian dynamics [44], [45]. Also, a valid assessment of spatiotemporal shifts in different functional types of vegetation might become essential to forecast feedbacks in stream flow changes and associated disturbance processes [20]. Although valuable, some of the latest approaches to riparian vegetation modeling still lack a spatial output of the functional type dynamics, which is essential for predicting and managing riparian ecosystems as a whole (e.g. [46], [47], [48], [49], [50], [51]).

In the present paper we endeavor to assess riparian vegetation structural changes caused by climate-changed flow regimes in different climatic and hydrogeomorphic contexts across Europe, as well as to consider responses to emerging topics that are yet insufficiently studied in fluvial ecosystems (see [52] for a better understanding), particularly with regard to riparian vegetation.

Preliminary results addressing such issues have been presented by the authors [53], [54], but not as comprehensively and using old-fashioned scenarios in some cases. The present work goes beyond the scope of those earlier results, inasmuch as it further analyzes riparian patch amendments in accordance with climate-driven hydrologic changes. Moreover, this is the first time that a joint effort to ascertain the spatiotemporal response of riparian ecosystems to climate-changed flow regimes, considering the latest climate change scenarios with available regional hydrologic forecasts and on a European scale basis, has been made.

## Methods

### Ethics Statement

This study was conducted on hydric public domain locations at the three considered countries. No specific permits were necessary for the described field studies as the performed observational assessments do not qualify as a procedure requiring a license under the national legislation of any of the mentioned countries. Field studies didn't involve elimination or removal of any endangered or protected species.

### Study site selection

**Figure 1:**
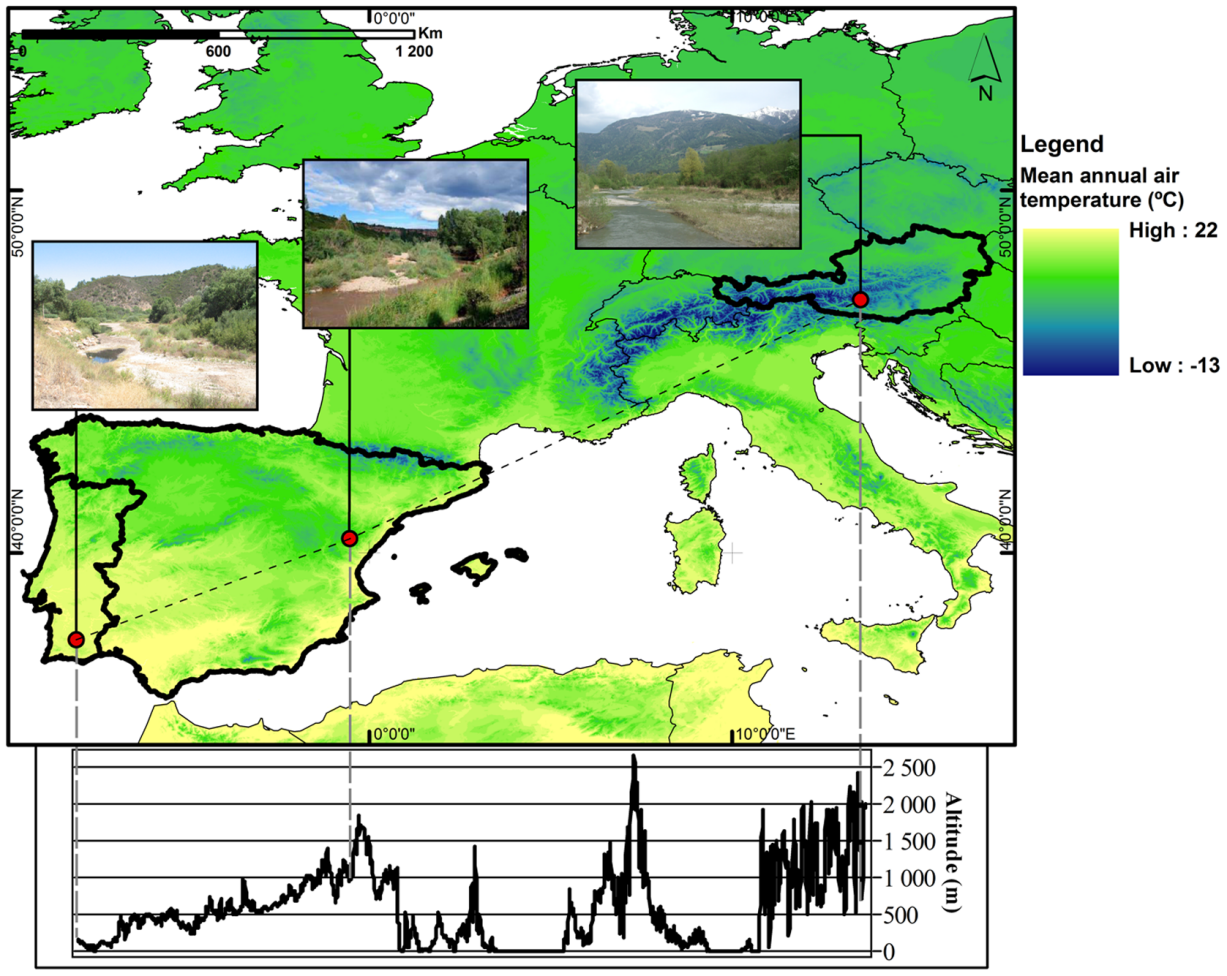
Study site location. Study site location showing the spatial variation in mean annual air temperature and an altitude profile across the three study sites (Digital Elevation Model and Mean annual air temperature data source: EDIT Geoplatform, [January, 2013], (CC BY-NC-SA 2.5 ES), http://edit.csic.es/).

Three study sites were selected with a view to encompass the principal watershed alimentation modes occurring across Europe. Although this was the primary criterion, we also attempted to consider an existing climatic gradient, determined by variables such as latitude, altitude or air temperature. Study sites (river reaches) were thus located in different countries with diverse climates and flow regimes (by both main water alimentation mode and transient pattern of discharge), namely Austria, Portugal and Spain (Figure 1).

#### Kleblach reach (Drau River, Austria)

**Figure 2:**
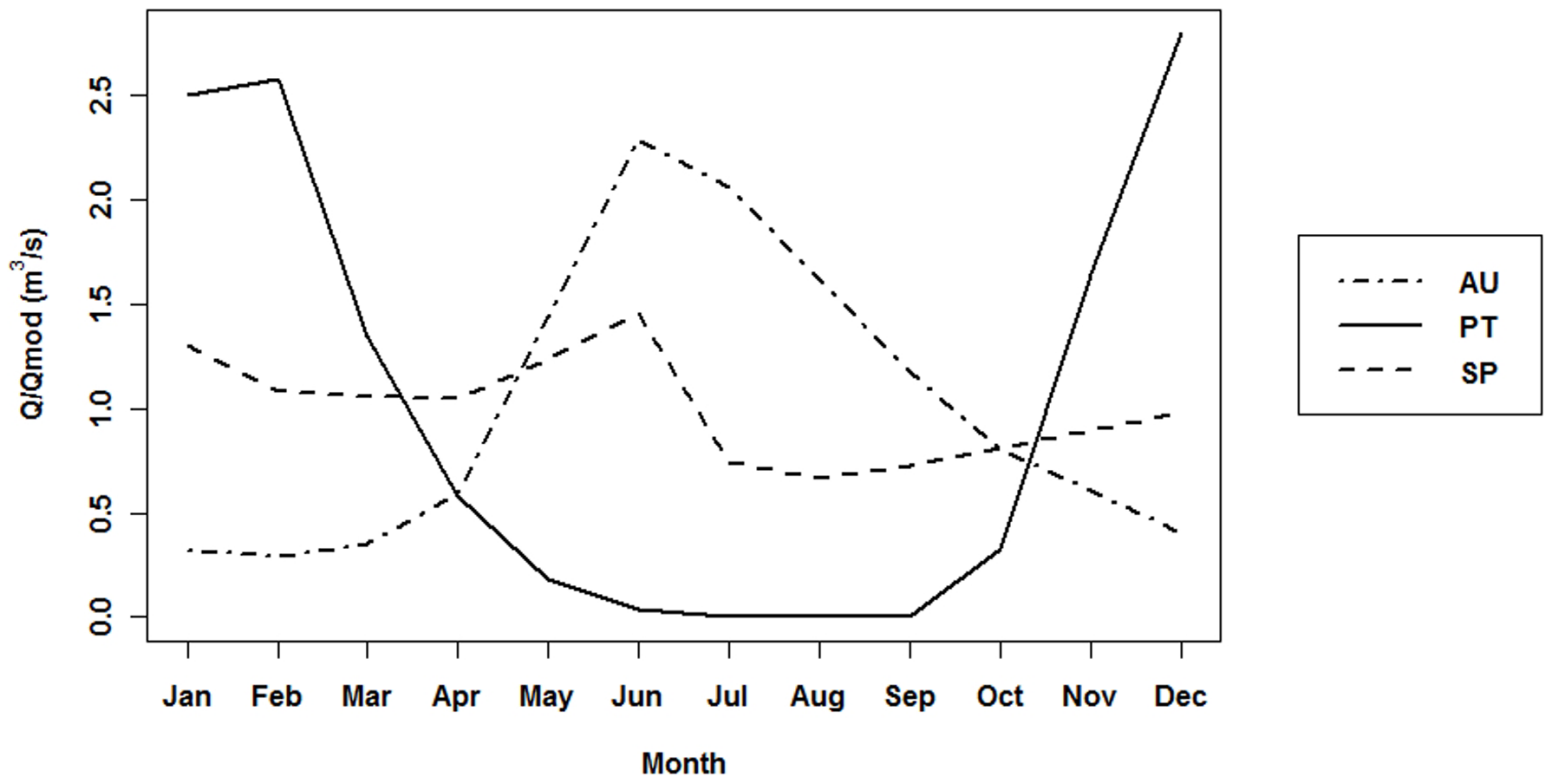
River flow regimes in the three considered study sites. River flow regimes in the three considered study sites (Austria – AU, Portugal – PT and Spain – SP). Mean monthly discharges are presented as ratio Discharge (Q)/Mean annual discharge (Q_av_) for 1960–1990 year period.

The Austrian case study is representative of the central Europe flow regimes, where maximum flows occur in spring and are attributable to snow-melt and glacial thaw. The study site is located at an altitude of approximately 570 meters in the upper river Drau, next to the village of Kleblach. Study site length is about 700 meters, and bank protection had been removed during an earlier river restoration project. Riparian vegetation comprises several species, most importantly including purple reed grass [*Calamagrostis pseudophragmites* (Haller f.) Koeler], German tamarisk [*Myricaria germanica* (L.) Desv], several willow species (*Salix triandra* L., *Salix purpurea* L., *Salix eleagnos* Scop. and *Salix alba* L.), grey alder [*Alnus incana* (L.) Moench] and European ash (*Fraxinus excelsior* L.). The river flow regime typifies a permanent temperate river, characterized by a mixed nivo-glacial regime [55] with significant flow (mean discharge between 1951 and 2008 equal to 74 m^3^/s) and a high degree of predictability. Although considered a mixed regime, only one real maximum occurs – in June-July, when the highest water levels occur as a result of watershed melt water flow-off. Conversely, minimum discharges occur in winter, due to solid precipitation and nival retention (Figure 2).

#### Ribeira reach (Odelouca River, Portugal)

The Portuguese case study exemplifies the South-Western Europe flow regimes, with minimum flows in summer due to the seasonal lack of rain. This study site is located in the Odelouca River, near Ribeira village, with a studied length of close to 400 meters, at an altitude of about 132 meters. Riparian vegetation is typically Mediterranean, inhabited mostly by tamarisk (*Tamarix africana* Poir.), willow (*Salix salviifolia* Brot.) and narrow-leaved ash (*Fraxinus angustifolia* Vahl.). In the outermost floodplain areas it is also possible to find the emergence among riparian species of terrestrial species like cork oak (*Quercus suber* L.) or holm oak (*Quercus ilex* L. subsp. ballota). This case study features an intermittent river with a simple pluvial regime, where maximum mean monthly discharges occur in winter, while minimum discharges (commonly null) take place in summer. River flow is generally low, but discharge is highly responsive to rainfall and flash floods happen whenever there are heavy rain events (although mean discharge is 2.5 m^3^/s, flash floods range between 80 and 480 m^3^/s). This hydrological regime thus displays a great intra and inter-annual variability (Figure 2).

#### Terde reach (Mijares River, Spain)

Typical river flow regimes of mountain-fed catchments are illustrated by the Spanish case study, located in the Mijares River, between the villages of Sarrión and Mora de Rubielos. This site lies at an altitude of approximately 850 meters, where it presents a permanent river, 540 meters of which were surveyed. The floodplain vegetation is generally characterized by different willow species (*Salix eleagnos* Scop., *Salix purpurea* L. and *Salix alba* L.), black poplar (*Populus nigra* L.) and common reed [*Phragmites australis* (Cav.) Trin. ex Steud.]. Terrestrial species like juniper (*Juniperus spp.*), kermes oak (*Quercus coccifera* L.) or holm oak (*Quercus ilex* L. subsp. ballota) are also found within the one hundred-year flood area. This case study is characterized by a mixed pluvio-nival river regime with a low mean monthly discharge coefficient amplitude. This river flow regime displays two mean monthly discharge maximums, one in January due to precipitation, and a more pronounced one in late spring originated by snowmelt (Figure 2).

### Climate change scenarios and expected hydrologic changes

In order to determine the deviation in riparian ecosystems caused by climate change, it is necessary to adopt a reference riparian patch mosaic from which to calculate riparian alterations linked to this stressor. To that end we considered a reference scenario, taking into account the popular and commonly used World Meteorological Organization (WMO) climate reference period of 1961–1990. This period is usually selected because it allows the comparison of future climate change regarding near present climatological conditions while having generally the best observational climate data coverage and availability from the periods considered meaningfully free from anthropogenic trends embedded [56].

The climate change scenarios adopted in this study were based on the latest IPCC emission scenarios from which hydrologic modeling have been performed. As described in its Special Report on Emission Scenarios (SRES) [57], this set of emission scenarios (A1 – *medium-high emission levels*, A2 – *high emission levels*, B1 – *low emissions* and B2 – *medium-low emissions*) attempts to reproduce the current knowledge in climate change science in order to characterize the range of probable driving forces and GHG emissions until 2100. Two of the above emission scenarios were selected for use as scenario templates in each case study, reflecting different intensities of climate change severity (Optimist and Pessimist scenarios) and spanning the existing uncertainties about future socioeconomic developments. In the light of the available data, the emissions scenario selection in each case study was determined in accordance with the Global and Regional Circulation Model scenarios whose results have been most consistent with the historical observations for each country, as regards temperature and rain forecasts in diverse climate change circumstances (see [58], [59], [60]). Corresponding discharge anomalies in the study site flow regimes were then obtained from national climate change assessments in which hydrology was also envisaged. The anomalies were applied to the existing reference flow regime data for each study site by multiplicative factors obtained in those studies to obtain the corresponding study site scenario data series.

As a result, for the Kleblach reach study site, SRES B1 and SRES A2 emission scenarios were selected as Optimist and Pessimist respectively. The GCM model used as a basis for these scenarios was GCM ECHAM5 [59]. The expected flow regime changes due to the projected meteorological alterations was determined by hydrological models based on information produced by the REMO-UBA regional climate model [59]. The climate change scenarios for the Ribeira reach were grounded in the RCM HadRM3 results for the Optimist SRES B2 scenario and the Pessimist SRES A2 scenario, as presented for Portugal by Santos *et al.* (2002, 2006) [58], [61]. The impact of climate change on freshwater assets was assessed using the Temez model – a simplification of the Stanford Watershed Model [62], [63]. Finally, for the Terde study site, the selected emission scenarios were also SRES B2 as the Optimist, and SRES A2 as the Pessimist. These were obtained from the Spanish modeling with the Hadley Centre Global Climate Model (HadCM3) as boundary conditions and regionalized with the PROMES regional climate model [60]. Hydrological scenarios were obtained from PATRICAL precipitation-runoff model results [64]. A summary of the hydrological changes considered for the aforementioned climate change scenarios for each study site is presented in Table 1.

**Table 1:** Hydrological regime modifications accounted for the riparian vegetation modeling in the considered climate changes scenarios.

		Austria		Portugal		Spain	
		Change SRES A2 (Pessimist)	Change SRES B1 (Optimist)	Change SRES A2 (Pessimist)	Change SRES B2 (Optimist)	Change SRES A2 (Pessimist)	Change SRES B2 (Optimist)
**Mean monthly discharge (%)**	**Winter (DJF)**	38	26	−60	30	−30	−27
	**Spring (MAM)**	9	12	−80	−25	−24	−25
	**Summer (JJA)**	−17	−12	−80	−50	−32	−27
	**Autumn (SON)**	−11	−3	−80	−60	−33	−30
**Minimum watertable elevation (m)**		NE	NE	−4	−1	−0.27	−0.25
**Flood duration**		NE	NE	NE	NE	NE	NE

Values stand for deviation from Reference period (1960–1990). NE stands for non-expected changes.

### Riparian vegetation modeling

For this task we used the state-of-the-art *Computer Aided Simulation Model for In-stream Flow and Riparian vegetation model*, commonly known as the *CASiMiR-vegetation* model [65]. This tool is a dynamic rule-based spatially distributed model that supports its computation on fluvial disturbance in riparian vegetation – a concept that has been increasingly recognized since the late 1980's [66] and whose influence is known to be a key cause of spatiotemporal variability in streams [27], [67], [68], [69], [70], [71]. More precisely, this tool relates ecologically relevant hydrological elements [17] with riparian vegetation features that directly respond to chronic hydrologic alteration [25], thus being able to reproduce local fluvial disturbance on an annual time step basis and determine the expected succession/retrogression phenomena in vegetation patches, depending on the fluvial physical driving forces to which they are subjected. The structure of *CASiMiR-vegetation* [65] consists of grid-based modules (*Recruitment*, *Morphodynamic disturbance* and *Flood duration*) functioning with a Boolean logic framed by hard thresholds derived from expert judgment. Together, those modules mimic the succession/retrogression episodes experienced by patches when subjected to a particular fluvial disturbance stress.

**Figure 3:**
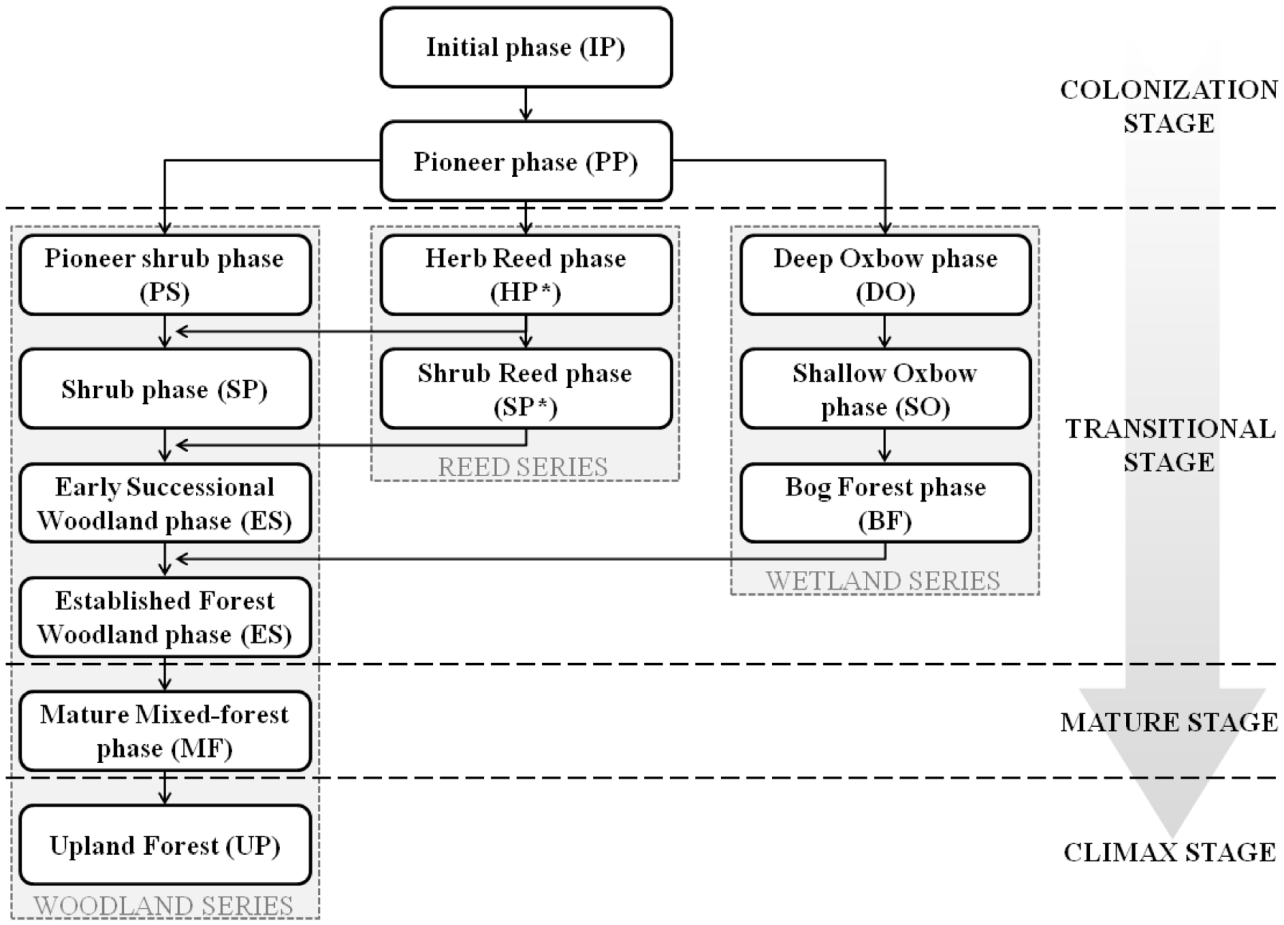
Common vegetation classification adopted for the three case studies. Common vegetation classification (by succession phase and stage) adopted for the three case studies, according to the existing vegetation series in each case study. Adapted from [74].

A huge asset of this model is that modeling is performed by succession phase instead of site-specific features. This permits worldwide application [53], [54], [72], [73], [74] and eliminates divergences (e.g. species composition, ecoregion differences) that make generalized application unfeasible in many other models (see [25]). Using this approach it is possible to obtain a homogeneous vegetation classification for the three case studies and thus permit a common appraisal of the modeling results. The adopted classification was first presented by García-Arias *et al.* (2013) [75] (see this reference for a more detailed explanation of the vegetation types/succession phases transformation process), in which thirteen succession phases embedded on four succession stages and three succession series were acknowledged (Figure 3).

With this classification the model presented substantial positive results at the calibration/validation stage and also proved that a study site comparison analysis using standardized succession phases is possible. In addition, model uncertainty due to estimation errors in estimated parameter thresholds was determined not to be significant [76]. *CASiMiR-vegetation* model calibration/validation for these cases is not presented here, as it is already thoroughly explained in previous studies [75], [77].

The input data needed to run this tool are grid-based topography, maximum annual discharge shear stress, flood duration and mean/base water table elevation files. Our topography inputs were obtained by topographic surveys and were considered to be fixed during the modeling runs, so that riparian change evaluation could be endorsed solely to the hydrologic regime changes. Shear stresses and water table elevations in each study site were obtained by 2D hydraulic modeling, while flood duration was retrieved from daily recorded discharge data [77]. Among the input data, shear stress stood out in terms of intra-scenario variability and was therefore analyzed for significant differences between scenarios. On the other hand, because minimum annual water table elevation and flood duration were considered unchanged within scenarios, we did not examine them by these means.

A simple method for appraising significant differences related to shear stress disturbance is to build confidence intervals for shear stress sample means in each scenario. We did this using two sample t-tests from the *R Stats package* in R environment [78].

Riparian vegetation modeling considered three modeling runs for each study site – namely the Reference, Optimist and Pessimist scenarios – starting from the same initial condition provided by the model. The expected 1990 riparian vegetation map was considered as the Reference scenario and was intended for use as a benchmark for assessing riparian deviations in the climate-change scenarios. The climate change scenarios (both Optimist and Pessimist) were characterized by the expected riparian vegetation maps at year 2100, under the corresponding climate-changed flow regimes. Once again, expected climate-changed riparian vegetation maps were obtained by modeling riparian vegetation under the likely river flow regimes in the 2071–2100 period. Riparian vegetation changes were analyzed by proportional change in total study site area and within each succession phase area, further denominated “specific area cover anomaly”, and referring to the difference between specific areas of succession phases in the Reference and climate-changed scenarios.

## Results

**Figure 4:**
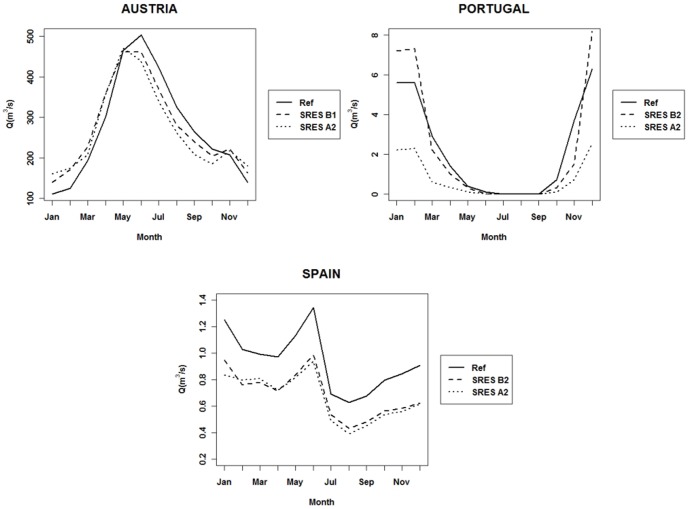
Reference and expected climate-changed hydrologic regimes in the considered study sites. Reference and expected climate-changed hydrologic regimes in the considered study sites (Discharge values stand for mean monthly discharges).

For all study sites, the expected flow regime in each climate change scenario follows a pattern similar to that of its reference regimes (Figure 4). Having said this, some changes are noticeable and can lead to structural modifications in riparian woodlands. In the Austrian case, both scenarios forecast similar changes in the hydrological regimes. Winter and early spring mean discharges are likely to be higher than those in the reference period, whereas in the remaining months mean monthly discharge is expected to be lower. Nonetheless, water table elevations and flood durations are not expected to change significantly (Table 1). In the Portuguese case study, changes in the flow regime differ depending on the climate change scenario. This discharge variability is found in winter, when river flows are expected to be higher in the Optimist scenario, but lower in the Pessimist one. In the remaining seasons, both scenarios predict a reduced discharge compared to the corresponding Reference scenario, which in turn will contribute to a water table drop of about 1 and 4 meters in the Optimist and Pessimist scenarios, respectively. No flood duration changes are expected in this flow regime, as floods occur on a very short period of time (Table 1). Finally, in the Spanish case study both scenarios show a decreased discharge throughout the hydrological year, with very similar changes. Fluvial disturbance is attenuated, and reduced water availability will be experienced in the floodplains all year long. Water table elevations are expected to decline about 0.25 m in the Optimist scenario and 0.27 m in the Pessimist, while no changes were predicted concerning flood duration (Table 1).

**Figure 5:**
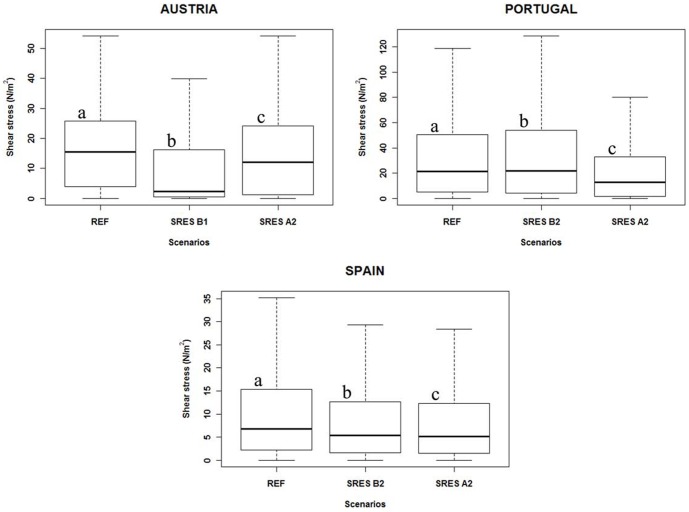
Scenarios of maximum annual discharge shear stress in each study site. Expected microhabitat shear stress of the maximum annual discharges in each study site according to the Reference, Optimist and Pessimist scenarios (whiskers stand for non-outlier extremes, box for 1st and 3rd quartiles, thick line for mean, and letters for significantly different groups).

Consistent with the expected climate change-induced flow regimes in each case study, maximum annual shear stress modifications in the study sites are also predicted. In fact, shear stress differences between scenarios proved significant with a 99% confidence level and corroborated earlier affirmations (Figure 5 and Table S1).

**Figure 6:**
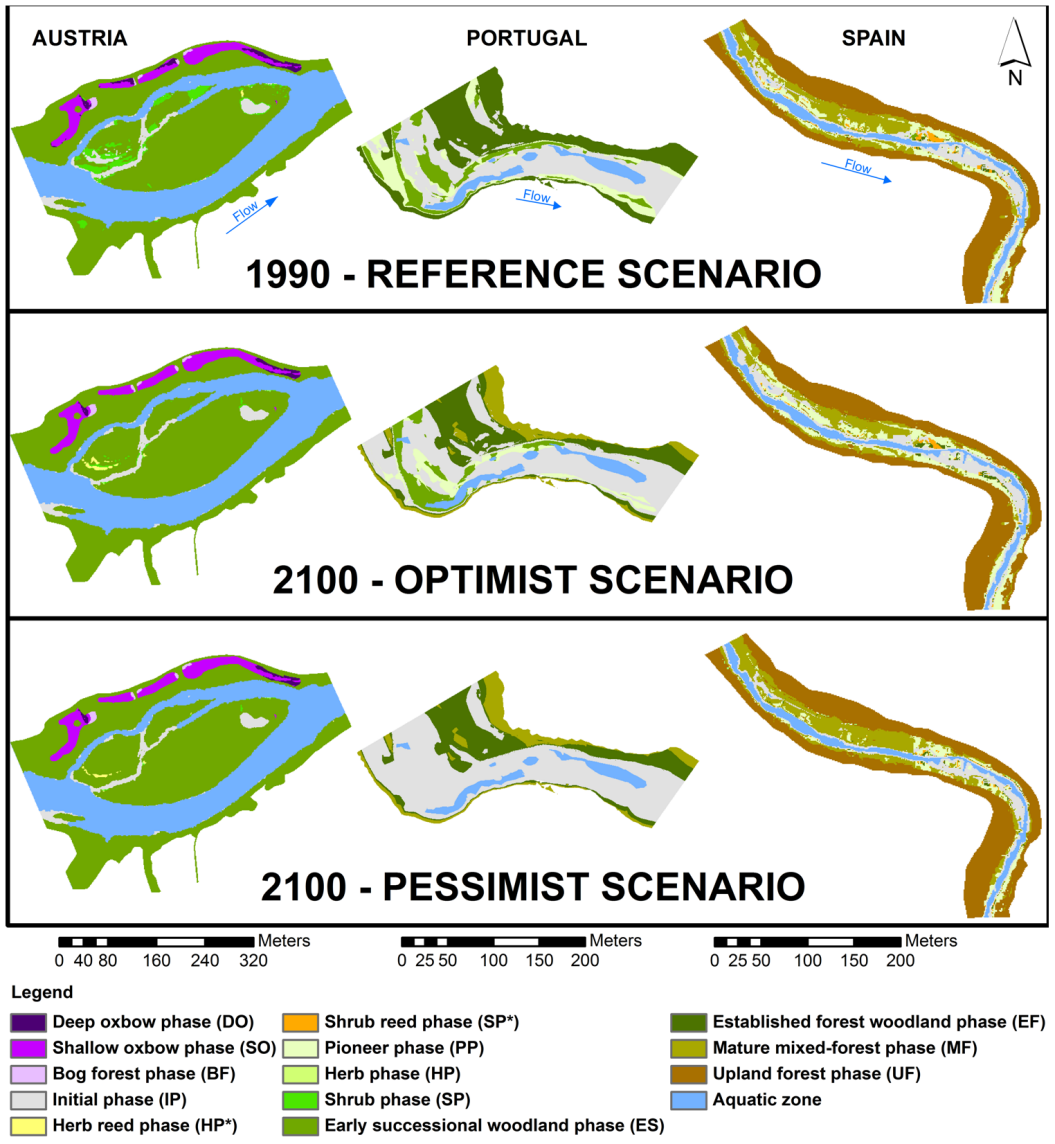
Riparian vegetation modeling results in each study site for the considered scenarios. Riparian vegetation modeling results in each study site for the Reference, Optimist and Pessimist scenarios.

Riparian vegetation modeling results show that, under the influence of climate-changed flow regimes, all the studied riparian ecosystems will experience structural changes in their riparian patch mosaics. Despite the fact that for the same modeling area (100-year flooded area), the three case studies achieved different stages in terms of vegetation development, the same tendency is perceptible in all of them. Novel succession phases are replaced by older and more hydric stress-tolerant ones in most cases; and wherever that replacement is not possible, riparian vegetation fades away, giving way to a complete retrogression to the Initial phase (Figure 6).

Table 2 illustrates the proportional area covered by succession phases in each study site scenario. Austrian Reference scenario is characterized by the existence of three different vegetation series, mostly in a Transitional Stage (approximately 95%) and with little Colonization stage (near 5%). Riparian corridor is composed mainly of Woodland series (almost 87% of total area), the most common phase being Early Successional Woodland (ES) with about 82% of total area. Wetland series cover around 8% of total study site area, with Deep Oxbow phase (DO) with 1.5%, Shallow Oxbow phase (SO) with nearly 6%, and Bog Forest phase (BF) with 0.5%. The Initial phase (IP) represents almost 5% of total study site area. In opposition to the Reference scenario, slight changes are predicted in succession phases. As an example, in both Optimist and Pessimist scenarios the Woodland series Shrub Woodland Phase (SP) converts into Early Successional Woodland Phase (ES) with a consequent decline of approximately 4% in total area. In the case of the Wetland series, despite maintaining its cover area in all modeled scenarios, its succession phases adjust towards improved hydric stress adaptation. In fact, in both climate change scenarios the Deep Oxbow Phase (DO) decreases by 0.7% of total area, in favor of the Shallow Oxbow Phase (SO), which increases by the same amount in both scenarios. Reed series appear in the form of the Herb Reed phase (HP*), taking over areas once occupied by the Initial phase (IP) and where fluvial disturbance previously precluded vegetation establishment. In a climate change scenario, this succession phase achieves a habitat settlement ranging from 0.2% (in the Pessimist scenario) to 0.4% (in the Optimist scenario) of the total study site area.

**Table 2:** Changes in succession phase cover area according to the considered scenarios.

			Austria					Portugal					Spain				
			Reference scenario	Optimist scenario		Pessimist scenario		Reference scenario	Optimist scenario		Pessimist scenario		Reference scenario	Optimist scenario		Pessimist scenario	
Succession series	Succession stage	Succession phase	%	%	Δ	%	Δ	%	%	Δ	%	Δ	%	%	Δ	%	Δ
**Any**	**Colonization stage**	**IP**	4.9	4.1	−0.8	4.1	−0.8	37.3	46.6	9.3	61.3	24.0	14.0	16.0	2.0	11.3	−2.7
**Any**	**Colonization stage**	**PP**	-	-	-	-	-	13.1	7.5	−5.6	0.0	−13.1	11.7	14.8	3.1	7.1	−4.6
**Woodland series**	**Transitional stage**	**HP**	-	-	-	-	-	-	-	-	-	-	0.1	0.0	−0.1	0.1	0.0
**Woodland series**	**Transitional stage**	**SP**	4.3	0.5	−3.8	0.1	−4.2	-	-	-	-	-	-	-	-	-	-
**Woodland series**	**Transitional stage**	**ES**	82.4	86.6	4.2	87.2	4.8	11.6	8.3	−3.3	0.0	−11.6	0.3	0.2	−0.1	0.1	−0.2
**Woodland series**	**Transitional stage**	**EF**	-	-	-	-	-	38.0	24.8	−13.2	25.8	−12.2	0.9	1.4	0.5	0.7	−0.2
**Woodland series**	**Mature stage**	**MP**	-	-	-	-	-	0.0	12.8	12.8	12.9	12.9	19.0	14.2	−4.8	23.1	4.1
**Woodland series**	**Climax stage**	**UF**	-	-	-	-	-	-	-	-	-	-	52.3	52.3	0.0	56.8	4.5
**Reed series**	**Transitional stage**	HP*	0.0	0.5	0.5	0.2	0.2	-	-	-	-	-	0.0	0.0	0.0	0.1	0.1
**Reed series**	**Transitional stage**	SP*	-	-	-	-	-	-	-	-	-	-	1.7	1.0	−0.7	0.7	−1.0
**Wetland series**	**Transitional stage**	**DO**	1.5	0.8	−0.7	0.8	−0.7	-	-	-	-	-	-	-	-	-	-
**Wetland series**	**Transitional stage**	**SO**	6.4	7.1	0.7	7.1	0.7	-	-	-	-	-	-	-	-	-	-
**Wetland series**	**Transitional stage**	**BF**	0.5	0.5	0.0	0.5	0.0	-	-	-	-	-	-	-	-	-	-

Succession phase changes (area cover) in accordance with the considered case studies and scenarios. See Figure 5 for succession phase acronyms; percentage values relative to the total modeling area in each case study; Δ stands for scenario variation when compared to the Reference scenario.

The Portuguese case study presents a Reference scenario composed of Colonization and Transitional stages, each occupying approximately half the total area. Succession phases are present in different proportions, with Initial phase (IP) and Established forest phase (EF) occupying the majority of the study area (nearly 40% of total area each). In the considered climate change scenarios, the increase in the Colonization stage is proportional to climate change severity, due to the retrogression of younger phases, which attain growth of more than 60% in the Pessimist scenario. On the other hand, the Transitional stage deviation takes an inverse route, with a reduction to 33% in the Optimist scenario, and to less than 26% in the Pessimist one. Considering the specificity of the succession phase, it is noticeable that all succession phases are expected to experience moderate changes, ranging from around 3 to 24% of total area. Initial (IP) and Mature Mixed-forest (MF) phases swell in both scenarios, with the former responsible for the biggest change in the riparian patch mosaic, specifically in the Pessimist scenario, where just this phase is responsible for a change in almost a quarter of the studied landscape. This increase occurs at the expense of the remaining phases, and even entails the total disappearance of the Pioneer (PP) and Early Succession Woodland (ES) phases in the worst scenario. The Established forest phase (EF) also reduces its cover area in the study site (roughly 13 and 12% in Optimist and Pessimist scenarios), but this time due to aging towards the Mature Mixed-forest phase (MF).

The Reference scenario in the Spanish study site is characterized by the existence of all the successional stages mentioned earlier and two succession series. Here, the Colonization, Transitional, Mature and Climax stages respectively comprise around 26, 3, 19 and 52% of the total area. With particular reference to the succession phases of the Woodland series, this case study is mainly represented by the Upland Terrestrial Forest (UF) and Mature Mixed-forest phases (together occupying nearly 70% of total area), while the remaining phases cover areas ranging 11% to 19% of the total area. Reed series cover almost 2% of the total area, namely in the form of a Shrub Reed Phase (SP*). In an Optimist climate change scenario, the Colonization stage increases by 5%, with a corresponding decrease in the Transitional and Mature Stages (0.4% drop-off in the former and nearly 5% in the latter). The Climax stage remains unaltered in this scenario. On the other hand, in the Pessimist scenario, Colonization and Transitional stages decline by approximately 7 and 1% of total area respectively, but the Mature and Climax Stages enlarge by approximately the same proportion of total area – namely 4% for the former and 4.5% for the latter. Where succession phases are concerned, minor changes are expected for the riparian patch mosaic in all the considered climate change scenarios, as none of the adjustments attain 5% of the total area. Major changes occur with the Pioneer (PP) and Mature Mixed-forest (MF) phases, but with no consistent trend. In fact, whereas in the Optimist scenario PP is expected to rise by nearly 3% and MF to decrease by about 5% of total area, in the Pessimist scenario PP faces a drop of almost 5%, but MF is reduced by more than 4% of total area. Changes in the Reed series represent a minute proportion of the total study area in both climate change scenarios.

**Figure 7:**
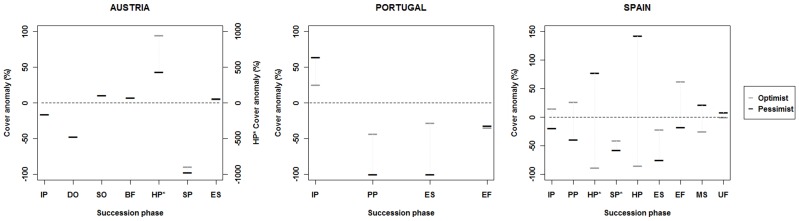
Specific area cover anomaly of succession phases. Specific area cover anomaly (%) of the succession phases in each study site and for the considered scenarios (see Figure 5 for succession phase acronyms).

However, although succession phase adjustments may not greatly change the riparian patch mosaic, standalone analysis does reveal profound alterations in the specific habitat area of each succession phase (Figure 7). This means that in the Austrian case some succession phases suffer extensive losses – e.g. the Shrub Woodland Phase (SP) experiences a specific decline in area of almost 90% in the Optimist scenario and faces near extinction (97.9% decline) in the Pessimist one. For the Wetland series, the Deep Oxbow Phase (DO) also faces a decrease in area of nearly 48% in both scenarios. In the Reed series there are noteworthy variations as well, but this time the Herb Reed phase (HP*) is expected to see a tenfold increase in area in the Optimist scenario and a fivefold one in the Pessimist scenario. In Portugal, at least a quarter of the areas of all the existing succession phases in the Reference scenario are expected to be modified in a climate change situation. The Pioneer (PP) and Early Successional Woodland (ES) phases are the most susceptible in this ecosystem, respectively suffering a specific area deprivation of approximately 43% and 28% in the Optimist scenario, while in the Pessimist scenario total retrogression may even occur. These decreases also lead to more than double the expansion of the Initial phase (IP) in the latter scenario. The Spanish case study is no exception to the other two, experiencing considerable succession phase changes. The area of Pioneer (PP) and Established Forest Woodland (EF) phases clearly increase, by almost 27 and 63% respectively in the Optimist scenario, while the Herb Woodland (HP) and Mature Mixed-forest (MS) phases are expected to suffer shrinkages in area of around 86 and 26% respectively. Succession phases in the Reed series are also prone to extensive reduction, with the Herb Reed (HP*) and Shrub Reed (SP*) phases losing roughly 89 and 41% of their specific areas. What is more, in the Pessimist scenario the Pioneer (PP) and Early Successional Woodland (ES) phases undergo a notable contraction in area of nearly 39 and 75% respectively. In this scenario the area of the Shrub Reed phase (SP*) is also likely to fall by approximately 58%, but it is estimated that the Herb Reed phase (HP*) will increase by almost 78%.

## Discussion

In all the considered cases there are expected changes in river flow regimes that can lead to significant effects on the hydraulic and hydrological conditions of riparian vegetation habitats, namely flood disturbance and hydric stress, which are effectively two of the most important conditioning factors in riparian dynamics [20], [79], [80], [81]. River regimes powered mainly by snow melt or glacial thaw will experience minor increases in discharge. Winter discharges will be higher due to less nival retention, whereas summer discharges will fall due to the depletion of snow storage and the resulting decrease in melt water. In river regimes where rainwater is the main form of water alimentation, there is some uncertainty with regard to winter months, as not all rain forecasts agree [7] and different flood disturbances are thus expected for this season, depending on the scenario. Nonetheless, both climate change scenarios expect riparian vegetation to be subjected to lower discharges and accentuated hydric stress in the remaining months of the year.

Accordingly, analyses of the microhabitat shear stresses of maximum discharges in each case study revealed significant differences between scenarios, proving that there will be a meaningful variation in flood morphodynamic disturbance in a climate change scenario.

The riparian vegetation modeling was performed using three different case studies contrasting in flow regime. Such flow regimes encompass the three main water alimentation forms of European rivers, according to Pardé's [82] and L'vovich's [83] typologies, recently upheld by Wrzesiński [84]. However, these case studies are representative of specific flow regime sub-types, which are not sufficient to make assumptions for the general trend of riparian vegetation changes driven by climate-changed flow regimes in Europe. Nevertheless, this study represents a first approach to portray that evolution.

To analyze the outcomes of the riparian vegetation model, one must regard a number of assumptions that first must be acknowledged. For this study, results should be understood within the context of vegetation patch dynamics, facing a certain scenario created by specific *CASiMiR-vegetation* model settings. The obtained forecasts need to be interpreted more as an indicative trend rather than an exact prevision, due to the shortcomings of modeling such a high dynamic and complex system. The model was calibrated for each basin, considering that the vegetation patches evolution is essentially conditioned by the maximum discharge and by the minimum water table elevation registered in each year. To forecast that for different climate change scenarios the maximum annual discharge series in each basin were multiplied by a factor and the water table elevations were changed, according to the literature considered for the climate change scenarios [58], [59], [60], [61], [64].

Despite the inherent stochasticity of fluvial systems, we opted by a deterministic modeling approach. Although, the non-consideration of the discharge sequence stochasticity of a flood event being a simplification, the maximum instantaneous discharge registered in each year seems to be the ultimate circumstance of morphodynamic forces driving the succession/retrogression dynamics of riparian woodlands (see [20], [27], [28], [79], [81], [85], [86]).

This restriction enhances the appreciation of broad features or general trends and allows the understanding on how specific components of the flow regime affect riparian vegetation (see [17] for a better understanding). Consequently, based on this deterministic approach, we were able to eliminate the response variability caused by flow regime stochasticity and thus be able to address riparian responses to the discharges that are really important to condition the riparian vegetation dynamics.

Moreover, the fact that our modeling approach considers a fixed topographic input between years obviously represents a simplification of the multifaceted complex fluvial processes occurring within the riverbed. The flow patterns occurring over the banks of a river with riparian galleries and movable bed are very complex and difficult to model with accuracy. The models that consider the movable bed are still relatively inaccurate, due to the use of different empirical formulas, and to the difficulty in obtaining the representative granulometric curve of the different sediment patches and of the different sediment layers of the river bed, not to mention the possible occurrence of layers armoring. In the same line, the hydrodynamic patterns through the riparian galleries are also very difficult to model, due to the vegetation heterogeneity and to the different bending resistance of vegetation species and of their succession phases to the flow velocity. The interaction between vegetation and sediment transport is, of course, still more complex. One example is the vegetation feedbacks, influencing the creation of fluvial landforms, trapping or stabilizing sediments, organic matter and the propagules of other plant species, i.e. acting as physical ecosystem engineers [87]. Another effect particularly relevant in these case studies is the retrogression of transitional and mature stages, which are retrogressed mainly by side erosion and bank failure rather than mechanical disturbance. This is an aspect that will be very difficult to model and that was not considered in the present research.

In this context, the authors believe these complex effects should not be considered, so that the obtained results can reflect the influence of the main succession driving factors: maximum annual discharge and minimum water table elevation.

Besides, despite the recent recognition of those issues concerning the modeling of interactions between flow regime, vegetation and morphology [88], [89], such processes were not yet implemented in the CASiMiR-vegetation model and would call for a specific research effort aiming at their integration in future model developments, not only within the climate change effects modeling but more generally within the riparian vegetation modeling context [88]. But, the development of suitable models to simulate and analyze the biogeomorphologic feedbacks is still a priority in ecogeomorphology science agenda [90], as limited capacity remains to predict flow properties in vegetated channels, due to the great difficulty of linking complex dynamic vegetation structures to non-homogeneous hydrogeomorphic processes [91]. Notwithstanding, in a similar study Politti *et al.* [92] suggested to consider a modeling period ranging from 5 to 25 simulated years, in order to work around those issues. According to this author, within this time frame the effect of the initial riparian landscape condition fades away after the 5^th^ year while the non-consideration of the river morphological changes is not relevant before the 25^th^ year.

Notwithstanding the previously stated, the performed vegetation modeling demonstrates that, for the considered flow regimes, contradictory changes are expected to occur in riparian ecosystems. While in snow-powered flow regimes succession is most likely to occur right across the transversal gradient of the river, in rain-fed watersheds a more complex situation is expectable, with retrogression prevailing inside the channel and succession occurring in areas further from the river. In typical river flow regimes fed by mountain catchments, greater changes will likely occur in the older phases of the ecological succession, but, as other authors have pointed out (e.g. [93], [94]), results are not linearly correlated to any of the imposed stresses. In fact, lower flood disturbance and increased hydric stress do not result in a clear tendency in riparian vegetation structural amendment terms, thus showing that in this case shear stress and hydric stress don't explain successional dynamics by themselves.

Nor is the extent of change equal across the considered flow regimes. In both nivo-glacial and mountain-fed flow regimes, moderate changes in total area do occur, but some particular smaller variations in certain succession phases may not be enough to say whether this adjustment is due to model causal effects rather than model uncertainty or input errors. In fact, such a detailed analysis should be conducted carefully as the average model area balance error of succession phases in the three case studies was about 7% [77], especially in smaller and highly disturbed patches like younger succession phases. On the contrary, in Mediterranean pluvial flow regimes, succession area changes can be substantial and rivers with flow intermittency seem to be the most affected [13], where succession phases can change *per se* almost a quarter of the total riparian patch mosaic.

Nonetheless, small changes in total area can mask dramatic habitat changes in succession phases within all the considered flow regimes. In fact, in the nivo-glacial regime-characteristic site, changes in succession phases can represent almost a tenth of the entire wetland areas, with large declines in some wetland succession phases, thus demonstrating that climate change will favor less water-dependent species. The same occurs in mountain-fed catchments, with succession phases experiencing specific area changes ranging from declines to near extinction, or to area boosts of about 50%. However, considering the variability of riparian responses to the climate-changed flow regimes in this case study, we are led to assume that in small river basins other factors may greatly influence riparian communities. These can include the availability of habitats provided by the river cross-section and the channel breath, or even human-related pressures [80], [95], [96].

All in all, climate-changed river flow regimes will most probably cause riparian vegetation amendments across rivers with similar flow regimes and even a general reduction in the areas covered by this vegetation. A common feature in all our case studies is that younger and more water-dependent phases are the most affected in a climate change scenario, whatever the forceful climate change or local environmental harshness may be. In snow-fed watersheds the main pathway for riparian vegetation appears to be succession, as minor summer floods cause less fluvial disturbance and greater hydric stress, which in turn allow vegetation to establish itself and develop to maturity, resulting in less water table-dependent phases. In pluvial flow regimes the tendency is consistently the opposite, despite some climatic uncertainties [7]. In this case, retrogression seems to be the main succession pathway for these communities, with large areas near river channels retrogressed to bare soil. Nevertheless, herein changes are not only due to the process of vegetation recycling to the Colonization stage, but also because of its aging to the Mature stage in the farthest floodplain areas. In mountain-fed catchments with mixed flow regimes the tendencies are not so clear and may reflect the existence of insufficient changes in flow regimes for there to be a clear change in their riparian communities. Meticulous analysis of the specific change in area in each succession phase showed that changes that may appear moderate when considering the total riparian patch mosaic can expose dramatic modifications when we look at the specific area changes in each succession phase. This means that many succession phases may face a serious threat in the future, when some of them will be confronted with complete annihilation. This outcome raises the question of maintaining viable populations of species that are important to conservation and are dependent on instream habitats. Additionally, more pronounced modifications – like the ones taking place in Southern European countries – are likely to occur in riparian communities that are dependent on pluvial flow regimes. These results are feasible expectations, inasmuch as similar riparian responses have been documented in vegetation assessments related to past flow regime events [20], [44], [48], [67], [97], [98], [99], [100]. There are also existing forecasts that support our findings [50], [60], [101], [102], [103], [104], [105], although generally more superficially and with less detail regarding inner riparian community structure diversity. Climate change can therefore endanger specific riparian species, drive shifts in which exotics become dominant [100], [106], or completely disrupt ecological succession in riparian ecosystems – something that can also lead to an increased risk to instream species survival [33] and flood hazards in downstream populations [36].

These results also pave the way for improved knowledge about emerging topics that are as yet insufficiently studied in fluvial ecosystems [52]. In this sense our results help substantiate the metacommunity Patch Dynamics Concept, which can be traced to Hutchinson's [107] seminal ideas about non-equilibrium communities, and reinforces the notion that competitively inferior species are favored by patch disturbance, without which they would be replaced by competitively superior ones. It also helps understand the effects of patch dynamics across different river gradients, as well as the fact that species' life history attributes can influence community dynamics in response to disturbed flow regimes and changed habitat characteristics.

Finally, the results obtained by us through vegetation dynamics simulation can generate new questions stemming from riparian ecology concepts. The expected changes in the spatial ratio of different riparian types, with the likely suspension of succession in some cases, could lead to reflection on the interplay between the fluvial setting and vegetation (e.g. [108]) – i.e. the relative dominance of non-equilibrium *versus* quasi-equilibrium processes [79]. Our work also suggests new scientific questions regarding the potential feedbacks of novel habitats associated with an altered riparian vegetation mosaic, leading to changes in shear stress disturbance and hydrogeomorphic processes [109], [110], or in relation to potential alterations in the global functioning of the ecosystem and thus the services it provides.

## Supporting Information

Table S1 **Confidence intervals for mean shear stress differences.** Confidence intervals for mean shear stress differences between scenarios in each case study.
(DOCX)
