# Supplementary material for: Modeling the Evolution of Riparian Woodlands Facing Climate Change in Three European Rivers with Contrasting Flow Regimes
Source: PLoS One. 2014 Oct 16;9(10):e110200. doi: 10.1371/journal.pone.0110200 (PMC4199630; doi:10.1371/journal.pone.0110200)
Supplement: Table S1 — Confidence intervals for mean shear stress differences. Confidence intervals for mean shear stress differences between scenarios in each case study. (DOCX) [file pone.0110200.s001.docx]

**Table S1. Confidence intervals for mean shear stress differences between scenarios in each case study.**

| **Case study** | **Reference – Optimist** | | **Reference – Pessimist** | | **Optimist – Pessimist** | |
| --- | --- | --- | --- | --- | --- | --- |
|  | **Confidence interval** | **p-value** | **Confidence interval** | **p-value** | **Confidence interval** | **p-value** |
| **Kleblach reach** | [6.38;6.61] | < 2.2e-16 | [1.69;1.92] | < 2.2e-16 | [-4.81;-4.57] | < 2.2e-16 |
| **Ribeira reach** | [-4.06;-2.11] | 3.248e-16 | [12.98;13.52] | < 2.2e-16 | [15.36;17.32] | < 2.2e-16 |
| **Terde reach** | [2.04;2.37] | < 2.2e-16 | [2.37;2.69] | < 2.2e-16 | [0.17;0.48] | 6.435e-8 |

Confidence intervals have a confidence level of 99%.
